# The regulatory role of the RANKL/RANK/OPG signaling pathway in the mechanisms of tooth eruption in patients with impacted teeth

**DOI:** 10.1186/s12903-020-01251-y

**Published:** 2020-09-18

**Authors:** Ludmila Brodetska, Larysa Natrus, Olha Lisakovska, Olexandr Kaniura, Liudmyla Iakovenko, Irina Skrypnyk, Petro Flis

**Affiliations:** 1 Department of Orthodontics and propaedeutics of Orthopedic Dentistry, Bogomolets National Medical University, Kyiv, Ukraine; 2 Research Institute of Experimental and Clinical Medicine, Bogomolets National Medical University, Kyiv, Ukraine; 3 Department of Biochemistry of Vitamins and Coenzymes, Palladin Institute of Biochemistry, Kyiv, Ukraine; 4 Department of maxillofacial surgery of childhood, Bogomolets National Medical University, Kyiv, Ukraine

**Keywords:** Impacted teeth, RANKL/RANK/OPG system, Nuclear factor kappa-B, Bone remodeling, Tooth eruption, Osteoclastogenesis, Apoptosis

## Abstract

**Background:**

Tooth impaction is a common problem in orthodontic practice and in some cases accompanied by pain and pathological changes of surrounding teeth. Understanding the cellular and molecular mechanisms underlying tooth impaction allows finding the most effective orthodontic treatment for patients with impacted teeth (IT). RANK (receptor activator of NF-κB) / RANKL (RANK ligand) / OPG (osteoprotegerin) signaling pathway controls bone resorption and may be involved in the regulation of tooth eruption. The study aimed to evaluate bone remodeling based on the assessment of the RANKL/RANK/OPG status in patients with IT.

**Methods:**

Bone samples from 18 patients (mean age 25.27 ± 3.34) were divided into 3 groups: 1 – bone tissue of healthy persons (control group); 2 – bone tissue, that was taken near the healthy tooth in patients with tooth impaction; 3 – bone tissue, that was collected near the IT. Levels of RANKL, RANK, OPG, osteocalcin (OC), NF-κB p65 subunit, NFATc1, and caspase-3 were determined by western blotting. The difference between groups was assessed using ANOVA followed by Tukey’s post-hoc test. *P*-value ≤0.05 was considered statistically significant.

**Results:**

We established a 1.73-fold elevation of RANK level in the IT area vs. control, indicating the recruitment of preosteoclasts. An increase in RANKL, OPG, and OC content was demonstrated (1.46-, 1.48-, and 1.42-fold respectively), reflecting the high activity of osteoblasts near the IT. Despite the activation of the RANKL/RANK/OPG system in the impaction area, NF-κB and NFATc1 levels did not change compared vs. control, indicating a blocked/delayed process of osteoclastogenesis. We found a decrease in the content of procaspase-3 (1.28-fold), while the level of its active form p17 increased by 2.26 folds near the healthy tooth in patients with IT compared with control. In the area of ​​IT, we observed an increase in procaspase-3 and p17 levels (1.32 and 1.78 folds). This reflects impairments of caspase-3 activation and accumulation of its inactive form in the IT area that may contribute to the tooth eruption failure.

**Conclusions:**

Tooth impaction may be associated with the disturbances in the caspase-3 cascade activation and the imbalance in the RANKL/RANK/OPG system, and as a result, blocked bone resorption.

## Background

Impacted teeth (IT) are those that remain partially or completely in the bone tissue or under the mucous membrane for 2 years after the term of their physiological eruption. The depth and direction of tooth impaction, and the amount of soft tissue or bone (or both) that covers teeth are diagnosed by X-ray examination. Tooth impaction is generally asymptomatic, and, in most cases, it is recognized by a chance in a routine dental examination, therefore patients seek treatment later than optimal. Nevertheless, in some cases, tooth impaction is accompanied by pain, pathological resorption of the surrounding teeth, and even an increased risk of malignancies [1]. Delayed or failed tooth eruption is a common problem in daily orthodontic practice; therefore, it is important to understand the cellular and molecular mechanisms underlying blocking tooth from breaking through the gum, to find the most effective orthodontic treatment for patients with IT. The presence of unerupted teeth requires a thorough diagnostics and a balanced approach to the choice of treatment method, which depends on the overall clinical situation, the patient’s age, and general somatic status.

The tooth eruption process is a highly complex regulated process in which the cells of both the tooth organ and the surrounding alveolus are involved. Tooth eruption is considered to be a valuable model for studying the process of bone remodeling since it involves tightly coordinated processes of bone formation and bone resorption with the participation of osteoblasts and osteoclasts respectively [2]. For an adequate eruption process, cells from the monocyte-macrophage lineage (osteoclast precursors) have to be recruited into the dental follicle during the pre-eruptive phase [3]. The process of their fusion occurs to form active multinuclear osteoclasts that are responsible for the formation of an eruptive pathway in the alveolar bone for tooth movement.

More recently, cytokine systems that are involved in the regulation of tooth eruption have been intensively explored, in particular, the main efforts have been focused on studying the signaling pathway RANK (receptor activator of nuclear factor kappa-B) / RANKL (RANK ligand) / OPG (osteoprotegerin).

RANKL is one of the tumor necrosis factor (TNF) superfamily members and acts through receptor RANK as a potent inductor of osteoclast formation and bone resorption. RANKL is produced by bone marrow stromal cells, osteoblasts, activated dendritic cells, and T-lymphocytes [4]. RANK is expressed as a transmembrane heterotrimeric receptor on the surface of haematopoietic osteoclasts progenitors, mature osteoclasts, chondrocytes, and other cells. Osteoprotegerin (OPG) is a decoy receptor for RANKL and prevents its osteoclastogenic action. The adequate balance between RANKL and OPG determines the rate of bone resorption [5]. An impaired OPG-to-RANKL ratio can be considered an essential pathogenetic factor for the development of bone tissue diseases.

RANKL/RANK/OPG axis regulates the maturation of osteoclasts and bone resorption, acting through the activation of the transcription factor NF-κB [6]. However, cells that are responsible for bone formation – osteoblasts – can influence the course of the tooth eruption by modulating the state of the RANKL/RANK/OPG axis as well, since they synthesize both osteoprotegerin, which is an inhibitor of bone resorption, and RANKL, which acts as its inducer. The role of the RANKL/RANK/OPG signaling pathway in the process of tooth eruption has been largely demonstrated on knockout rodents [7–9]. It has been shown that RANK and RANKL are expressed in dental follicles during tooth eruption and in periodontal ligament during adulthood [10]. OPG is a factor, important for homeostatic control of periodontal ligament functioning [11] and protects the cementum against root resorption [12]. Nevertheless, there is a lack of experimental studies related to the role of the RANKL/RANK/OPG system in the tooth eruption process in humans due to the difficulties with obtaining the written permissions from patients and approval from the local ethics committee.

Understanding the cellular and molecular mechanisms underlying tooth eruption and its complications allows developing and implementing a most effective orthodontic treatment for patients with impacted teeth in clinical practice. Given this, the aim of the study was to evaluate the status of bone remodeling based on the assessment of changes in the RANKL/RANK/OPG axis in patients with tooth impaction.

## Methods

### Ethical approval

The study was registered with the research center of Bogomolets National Medical University. Ethical approval for the study was obtained from the Ethics and Research Committee of Bogomolets National Medical University (Protocol No 95 from 05/05/2016). Written informed consent was obtained from each patient. Any uncooperative patients and patients who had not provided consent to participate in the study were excluded.

### Patients and sample collection

A total of 18 participants were analyzed. Calibrated dental hygienists performed whole-mouth oral examinations before orthodontic treatment. Table 1 shows patient’s characteristics including age, gender, type of orthodontic treatment, and the principle оf group formation. Among 18 participants, the gender distribution was as follows: 9 males (25.44 ± 3.43) years and 9 females (25.11 ± 3.06). The inclusion criteria of patients in the control group were the obligatoriness to remove аn erupted third molar and orthodontic prescriptions for the installation of the stainless steel brackets simultaneously. The inclusion criteria in the tooth impaction group were the presence of tooth impaction (maxillary third molars) and orthodontic prescriptions for installation of the stainless steel brackets. The same brand and model of stainless steel brackets (Dentaurum, Germany) were used for all patients. Bone samples from control patients and patients with tooth impaction were divided into three groups: 1 – bone tissue of healthy persons from the area of the normally eruthird molar that has been removed according to orthodontic prescriptions (control group); all teeth were normally erupted in patients of this group (based on the data of X-ray diagnostics); 2 – bone tissue of patients with tooth impaction, that was taken from the area of normally erupted third molar that has been removed according to orthodontic prescriptions; 3 – bone tissue of patients with tooth impaction, collected near the impacted third molar.

**Table 1:** Characteristics of 18 participants of the study (Kyiv, Ukraine) (*n* = 18, M ± m)

Patients	Group	Patient №	Age range	Maxillary/ mandibular	Type of orthodontic treatment	Weight of bone tissue sample, g
Patients with normally erupted teeth	#1 – bone tissue, that was taken from the area of the normally erupted third molar (control group)	1	21–28 Mean ± SD*25.00 ± 3.03*	maxilla	Metal orthodontic brackets	0.0457
		3		maxilla	Metal orthodontic brackets	0.0522
		4		maxilla	Metal orthodontic brackets	0.0674
		5		maxilla	Metal orthodontic brackets	0.0575
		6		maxilla	Metal orthodontic brackets	0.0661
		8		maxilla	Metal orthodontic brackets	0.0355
Patients with tooth impaction	#2 – bone tissue of patients with tooth impaction, collected from the area of the normally erupted third molar	9	21–29 Mean ± SD*25.42 ± 3.34*	maxilla	Metal orthodontic brackets	0.0341
		10		maxilla	Metal orthodontic brackets	0.0481
		11		maxilla	Metal orthodontic brackets	0.0452
		12		maxilla	Metal orthodontic brackets	0.0503
		13		maxilla	Metal orthodontic brackets	0.0388
		14		maxilla	Metal orthodontic brackets	0.0426
	#3 – bone tissue of patients with tooth impaction, collected from the area of the impacted third molar	15		maxilla	Metal orthodontic brackets	0.0513
		16		maxilla	Metal orthodontic brackets	0.0641
		17		maxilla	Metal orthodontic brackets	0.0852
		18		maxilla	Metal orthodontic brackets	0.0374
		19		maxilla	Metal orthodontic brackets	0.0371
		20		maxilla	Metal orthodontic brackets	0.0509

The surgical approach was used to collect bone samples near the maxillary third molars from the control group. Under the tuberal and greater palatine nerve block (sol. Ubistesin 4%, 2 ml) the third molar was removed by the traditional method. During the inspection, free bone tissue fragments of the alveolar socket were collected. To take the samples of bone tissue from patients with tooth impaction a surgical approach was used as well. After infraorbital and nasopalatine nerve block (sol. Ubistesin 4%, 2 ml) an incision of the mucous membrane was carried out followed by separation of the mucoperiosteal flap. The bone fragment that covered (or partially covered) the crown of the tooth was removed (osteoectomy) by bone nipper in the area of the impacted tooth. After collection bone samples were frozen in liquid nitrogen to prepare total protein lysates.

### Western blot analysis

Levels of RANKL, RANK, OPG, osteocalcin (OC), NF-κB p65 subunit, NFATc1, and caspase-3 in bone tissue were determined by western blot analysis. Bone tissue samples were ground in a porcelain mortar with liquid nitrogen. After that samples were lysed by an incubation on ice for 20 min in the appropriate volume of RIPA buffer 1:6 weight-to-volume ratio (20 mM Tris-HCl, pH 7.5; 150 mM NaCl; 1% Triton X-100; 1 mM EGTA; 0.1% SDS; 1% sodium deoxycholate; 10 mM sodium pyrophosphate) containing protease inhibitor cocktail (PIC, Sigma, USA), sonicated and then centrifuged at 14000 g for 20 min at + 4 °C. Protein concentrations in the lysates were measured by Lowry’s method. 30 μg of protein were loaded onto 10% SDS polyacrylamide gels. After SDS-PAGE, the proteins were transferred onto a nitrocellulose membrane (350 mA, 1 h). Immunoblotting was conducted with primary antibodies against RANKL and OPG (1:250; Santa Cruz, USA), RANK, OC and caspase-3 (1:500; Santa Cruz, USA), NF-κB p65 subunit (1:500; Thermo Fisher Scientific, USA), NFATc1 (1:1000; Thermo Fisher Scientific, USA) and β-actin (1:10000; Sigma-Aldrich, USA). Primary-antibody-bound membranes were incubated with peroxidase-conjugated secondary antibodies: anti-mouse IgG (Fab Specific)-Peroxidase (1:2500; Sigma, USA), anti-rabbit IgG (H + L)-HRP conjugate (1:4000; Bio-Rad Laboratories, Inc., USA) or anti-goat IgG (H + L) (1:2500; Invitrogen, USA). Thereafter the membranes were developed with chemiluminescent agents: p-coumaric acid (Sigma-Aldrich, USA) and luminol (AppliChem GmbH). Tissue levels of all proteins were normalized to β-actin. The immunoreactive bands were quantified using Gel-Pro Analyzer32 software.

### Statistical analysis

The results are expressed as a mean ± SEM. The hypothesis of normality distribution of data was tested by the Shapiro-Wilk test. Statistical differences among the means between groups were analyzed using the analysis of variance (ANOVA) and Tukey’s post hoc test. Differences were considered to be statistically significant when the *p*-value was less than 0.05. All statistical analyses were carried out using Origin Pro 8.5 (OriginLab Corporation, Northampton, MA, USA).

## Results

### Assessment of the RANKL/RANK/OPG axis and a ratio between its components in bone tissue of patients

**Fig. 1:**
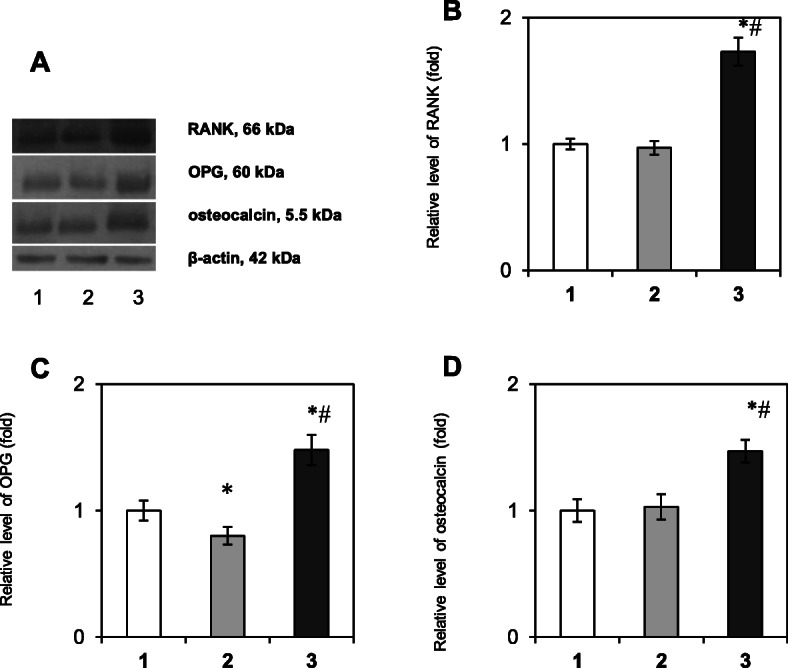
Immunoblotting analysis of RANK, OPG, and osteocalcin in bone tissue samples: representative immunoblots are shown (**a**) and quantified using β-actin as a loading control. The bar graphs of RANK (**b**), OPG (**c**) and osteocalcin (**d**) are presented as arithmetic means ± SEM (*n* = 6/group): 1 – bone tissue of healthy persons (control group); 2 – bone tissue, that was taken near the healthy tooth in patients with tooth impaction; 3 – bone tissue, collected near the impacted tooth. Experiments were performed in triplicate and *p < 0.05 denotes significance compared with control, #*p* < 0.05 denotes significance compared with bone tissue taken near the healthy tooth in patients with tooth impaction

**Fig. 2:**
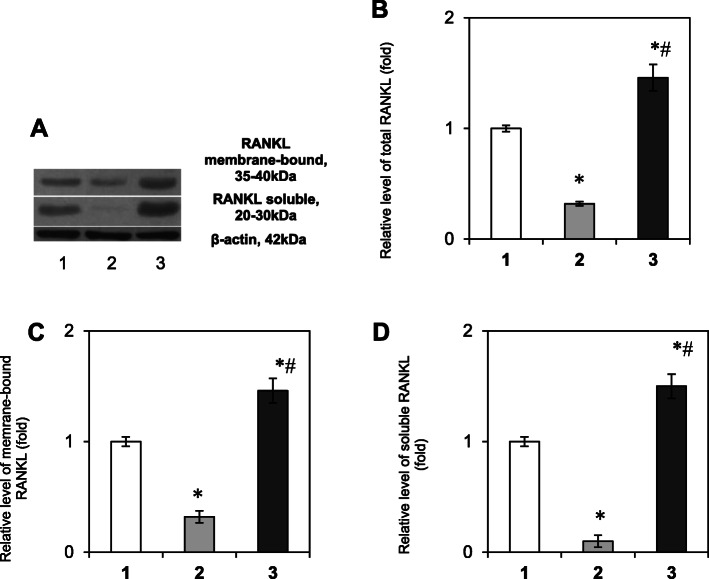
Immunoblotting analysis of RANKL in bone tissue samples: representative immunoblots are shown (**a**) and quantified using β-actin as a loading control. The bar graphs of the total content of RANKL (**b**), its membrane-bound (**c**) and soluble (**d**) forms are shown as arithmetic means ± SEM (*n* = 6/group): 1 – bone tissue of healthy persons (control group); 2 – bone tissue, that was taken near the healthy tooth in patients with tooth impaction; 3 – bone tissue, collected near the impacted tooth. Experiments were performed in triplicate and *p < 0.05 denotes significance compared with control, #*p* < 0.05 denotes significance compared with bone tissue taken near the healthy tooth in patients with tooth impaction

As the RANKL/RANK/OPG signaling axis regulates the interplay between osteoclasts and osteoblasts that can influence bone remodeling [6], we first determined the content of the RANKL/RANK/OPG system components in the bone tissue of patients with tooth impaction. The results of the study presented in Fig. 1 show that there is no difference in the relative protein content of RANK (Fig. 1 a, b, Tukey’s test, *p*-value > 0.05) and osteocalcin (Fig. 1 a, d, Tukey’s test, *p*-value > 0.05) between the samples of healthy persons (control group) and samples, that were taken near the normally erupted third molar in patients with tooth impaction. At the same time, it was shown a slight tendency to decreased content of osteoprotegerin (by 1.2 times) in the bone tissue near the healthy tooth in patients with tooth impaction compared with the control (Fig. 1c, Tukey’s test, *p*-value 0.048) and we observed a significant decrease in the RANKL content (membrane-bound form – by 1.6 folds, Tukey’s test, *p*-value 0.021, and soluble form – by 10 folds, Tukey’s test, *p*-value 0.003) (Fig. 2c, d).

Completely different results were obtained when studying the area of ​​the impacted third molars. Western blot analysis data revealed a 1.73-fold elevation of the RANK level in the IT area vs. control (Fig. 1b, Tukey’s test, *p*-value 0.0008), indicating an accumulation of osteoclast precursors. Moreover, a significant increase in the relative content of OPG (Fig. 1c, Tukey’s test, *p*-value 0.012), osteocalcin (Fig. 1d, Tukey’s test, *p*-value 0.021) and total RANKL (Fig. 2b, Tukey’s test, *p*-value 0.009) proteins (by 1.48, 1.42, and 1.46 folds, respectively) was demonstrated as well, that could correlate, at least partially, with the high activity of osteoblasts and/or its precursors in the area of IT.

Physiological effects of OPG and RANKL on bone tissue metabolism are known to be largely determined by the ratio of their synthesis [13]. Based on the measurement of protein levels of RANKL/RANK/OPG signaling pathway components by western blot analysis, we obtained the ratios applied for assessing the status of local bone remodeling in the areas near the healthy and impacted teeth in patients with tooth impaction. It was established a significant decrease in the RANK/RANKL (2.6-fold) ratio and an increase in RANKL/OPG (2.5-fold) ratio in the area near the impacted tooth compared with the healthy tooth (Table 2). Declined RANK/RANKL ratio in the IT area may reflect the disturbances in the ligand-to-receptor binding and indicate the reduced effectiveness of signal transduction in the RANKL/RANK/OPG axis in addition to elevated RANKL/OPG ratio. Based on these observations we can assume that the local interrelation between bone resorption and bone formation in the IT area may lead to delayed tooth eruption/tooth impaction.

**Table 2:** Ratios of the RANKL/RANK/OPG signaling pathway components in the bone tissue of patients, in a.u. (arbitrary units)

Ratio, a.u.	Bone tissue, collected near the healthy tooth in patients with tooth impaction	Bone tissue, collected near the impacted tooth
RANK	0.97 ± 0.06	1.73 ± 0.11*
RANKL	0.32 ± 0.02	1.46 ± 0.12*
OPG	0.8 ± 0,07	1.48 ± 0.13*
RANK/RANKL	3.03	1.18*
RANKL/OPG	0.4	0.99*

* *p* < 0.05 vs. bone tissue taken near the healthy tooth in patients with tooth impaction

### NF-κB-mediated signaling and apoptosis in the bone tissue

Binding RANKL to RANK leads to the further activation of NF-κB and translocation of this transcription factor to the nucleus to stimulate osteoclastogenesis [14]. To address whether excessive RANKL and RANK levels in bone tissue near the area of tooth impaction are engaged in the downstream transcriptional activation, we explored the involvement of NF-κB in the mechanisms of tooth eruption.

**Fig. 3:**
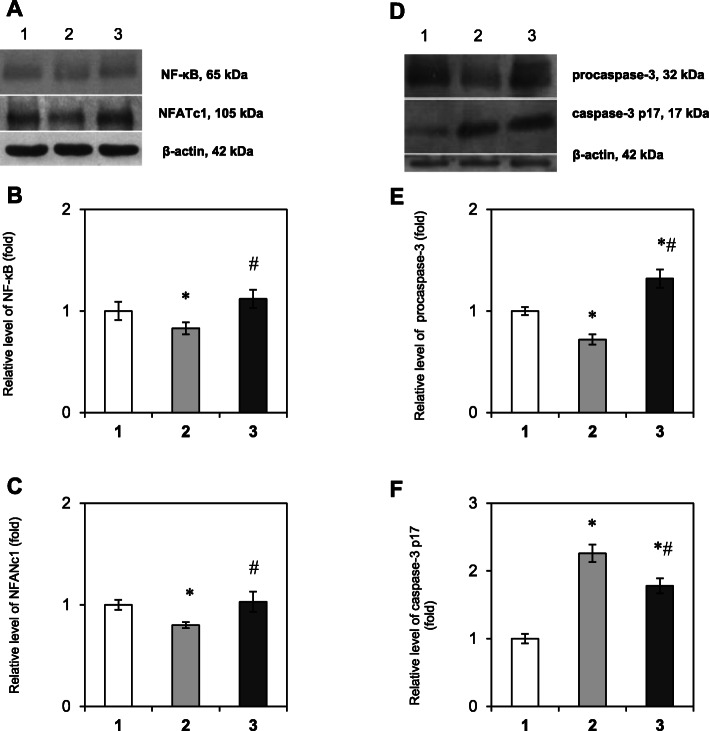
Immunoblotting analysis of NF-κB, NFATc1, and caspase-3 in bone tissue samples: representative immunoblots are shown (**a, d**) and normalized to β-actin. The bar graphs of NF-κB (**b**), NFATc1 (**c**), procaspase-3 (**e**) and its active subunit p17 (**f**) are demonstrated as arithmetic means ± SEM (*n* = 6/group): 1 – bone tissue of healthy persons (control group); 2 – bone tissue, that was taken near the healthy tooth in patients with tooth impaction; 3 – bone tissue, collected near the impacted tooth. Experiments were performed in triplicate and *p < 0.05 denotes significance compared with control, #*p* < 0.05 denotes significance compared with bone tissue taken near the healthy tooth in patients with tooth impaction

We demonstrated a slight decrease in the NF-κB (Fig. 3a, b, Tukey’s test, *p*-value 0.043) and NFATc1 (Fig. 3a, c, Tukey’s test, *p*-value 0.038) levels by 17 and 20% respectively in the bone tissue selected near the healthy tooth compared vs. control patients. Interestingly, it was demonstrated for the first time that despite the activation of the RANKL/RANK/OPG system in the impaction area, the content of both NF-κB and NFATc1 did not significantly change compared with the control group (Tukey’s test, *p*-value > 0.05), indicating a blocked or delayed process of osteoclastogenesis near the IT although an accumulation of osteoclast precursors in this area was observed.

Since caspase cascades play a key role in the activation of cell apoptosis [15] and may be triggered by NF-κB, we determined the levels of procaspase-3 (32 kDa) and its active subunit p17 (17 kDa). It was shown a decrease in the content of procaspase-3 (32 kDa) in the area of ​​a healthy tooth of patients with tooth impaction by 28% (Tukey’s test, *p*-value 0.031), while in the area of impacted tooth its content was increased by 1.32 folds compared with control (Fig. 3e, Tukey’s test, *p*-value 0.028). Сonsidering the active caspase-3 enzyme is a heterodimer consisting of two p17 and two p11 caspase-3 subunits [16], it was also important to evaluate the content of the activated p17 form. We detected an increase of its protein level in the area of the healthy tooth by 2.26 folds compared vs. control (Tukey’s test, *p*-value 0.0007), and an elevation in the area of the impacted tooth by 1.78 folds (Fig. 3f, Tukey’s test, *p*-value 0.001) on the background of an accumulation of inactive procaspase-3 32 kDa. These observations indicate the cleavage of procaspase-3 and its activation in the area of the healthy tooth of patients with IT, that promote the tooth eruption process. We also suggest the impairment of caspase-3 activation and the accumulation of an inactive form in the area of tooth impaction may lead to tooth eruption failure.

Our data demonstrate that tooth impaction may be associated with blocked NF-κB signaling and NFATc1 expression and with the disturbances in the cascade activation of caspase-3.

## Discussion

The cellular and molecular mechanisms underlying tooth impaction remain poorly understood. It is assumed that an imbalance between the osteosynthesis and bone resorption is one of the factors playing an important role in the development of tooth eruption abnormalities [17]. A key role in controlling the process of bone remodeling belongs to cytokine signaling systems. The regulatory protein osteocalcin, which is produced by osteoblasts [18], is involved in the process of osteosynthesis and considered a valid marker of bone formation. In turn, bone resorption is regulated by the osteotropic system RANKL/RANK/OPG [5].

The role of the RANKL/RANK/OPG signaling pathway in tooth eruption has been demonstrated in animal models to a greater extent [19]. Nevertheless, there is a lack of experimental studies dedicated to the role of the RANKL/RANK/OPG axis in the tooth eruption process in humans under the normal and pathological conditions due to the complexity of sampling and obligatoriness of confirmation from patients and ethics committee approval. The small number of studies dealing exclusively with the changes in the state of the cytokine system RANKL/RANK/OPG in bone tissue of patients with impacted teeth is available [20]. Therefore, the purpose of our research was to determine the levels of its key components as indicators of the processes of bone resorption and bone formation.

The current findings showed no significant difference in RANK and OC levels between the bone tissue samples of healthy persons and samples taken near the healthy tooth in patients with tooth impaction. A slight tendency to decreased OPG level in the bone tissue near the healthy tooth in patients with tooth impaction compared with the control was observed on a background of a significant decrease in the content of membrane-bound and soluble forms of RANKL. These findings clearly indicate a slight deviation in the production of cytokines by osteoblasts. Nevertheless, since we demonstrated a simultaneous decrease in the content of both cytokines RANKL and OPG on the background of the unchanged level of the preosteoclast marker RANK and marker of osteosynthesis osteocalcin, in general, this ratio of locally synthesized cytokines ensured a normal process of tooth eruption.

Interestingly, we obtained completely different results concerning the state of the RANKL/RANK/OPG system in the area of impacted tooth. The findings of the present study demonstrated a significant increase in the relative content of RANK, OPG, both forms of RANKL and osteocalcin in the IT area vs. control. Based on these observations we can assume an accumulation of osteoclast precursors as well as active osteoblastic cells, which excessively produce cytokines in the area of the impacted tooth. In addition, since RANKL is produced not only by osteoblasts, but by activated by B and T cells as well [21], high RANKL level may be explained by the recruitment of immunocompetent cells and the development of the local proinflammatory reaction near the impaction area – the formation of mononuclear infiltrations, accumulation of preosteoclasts, preosteoblasts and fibroblasts, that actively produce cytokines. Nevertheless, despite the high levels of cytokines tooth eruption does not occur in this area.

Therefore, it may be important to consider the ratios of the components of the RANKL/RANK/OPG system that are determined its physiological effects on the bone tissue metabolism. We used two indices for assessing the state of local bone remodeling in the areas near the healthy and impacted teeth in patients with tooth impaction: RANK/RANKL mainly as an indicator of bone resorption and RANKL/OPG that reflects the balance between bone formation and resorption. In the area of the impacted tooth, we found a decreased RANK/RANKL and increased RANKL/OPG ratios compared with the healthy tooth that may indicate impaired bone remodeling process and, as a result, disturbances in tooth eruption process. Moreover, it has been reported earlier an increase in RANKL/OPG ratio in periodontal tissues under inflammatory pathological conditions, such as periodontitis [22]. This confirms our assumption that an elevated RANKL/OPG ratio also indicates the development of the local proinflammatory reaction near the area of impacted teeth that may lead to the manifestation of such symptoms as pain and swelling.

This method, based on comparison of the RANK/RANKL and RANKL/OPG ratios during the normal and pathological eruption process, allows to estimate the intensity of the processes of bone resorption and bone formation in patients and may be recommended for implementation in orthodontic practice for the detection of local cytokine dysregulation in the impaction area and for choosing an effective method for correction of this pathological condition.

In the last decades, considerable progress has been made in understanding the molecular basis of osteoclastogenesis. It has been discovered that the process of osteoclast formation requires the involvement of NF-κB signaling, which is activated in response to several major “osteoclastogenic” cytokines, in particular, RANKL and macrophage colony-stimulating factor 1 (M-CSF1) [23]. RANKL and other proinflammatory cytokines such as tumor necrosis factor alpha (TNFα), activate NF-κB-associated signaling pathways, inducing c-Fos and nuclear activated T-cell factor (NFATc1), and also inhibit NFATc1 repressors, thereby positively regulating formation and activation of osteoclasts [24].

In addition to the importance of the balance between osteosynthesis and bone resorption, it is known that reorganization into the periodontal membrane is also required for an adequate course of tooth eruption [25]. This process involves the activation of apoptosis of the inner cell layer of the periodontium and the formation of a moving layer of apoptotic cells. Recently, it has been suggested that inappropriate process of apoptosis of osteoblasts/osteocytes accounted for, at least in part, the imbalance of bone remodeling. Accumulated evidence suggests that caspase-3 is a critical enzyme for apoptosis and cell survival; however, the exact role of caspase-3 in bone remodeling and development of skeletal diseases is largely unknown. To clarify why despite the high osteoclastogenic potential of cells in the impaction area, tooth eruption does not occur, the next step was to estimate the state of NF-κB signaling as a trigger factor for apoptosis in bone tissue of patients with impacted teeth.

Since the RANKL level was declined in the bone tissue selected near the healthy tooth of patients with tooth impaction compared with control patients, it was logical to observe a slight decrease in the NF-κB level and, as a result, in the NFATc1 and procaspase-3 (32 kDa) levels. Nevertheless, the content of caspase-3 subunit p17 was significantly higher thereby ensuring the reorganization into the periodontal membrane and normal course of the tooth eruption.

In the present study, we found that there was no difference in the NF-κB and NFATc1 levels compared with the control group despite the established activation of the RANKL/RANK/OPG signaling axis in the impaction area. Although an accumulation of osteoclast precursors in the IT area was observed, our results indicate a blocked or delayed process of osteoclastogenesis in the bone tissue near the impacted tooth. These data indicate that there is no adequate maturation and activation of osteoclasts, therefore resorption in the area of the impacted tooth does not normally occur, which may explain the tooth impaction and/or delayed tooth eruption. Understanding the molecular basis of impaired bone resorption in the IT area is required for further investigation.

While assessing the process of apoptosis we detected an increase in caspase-3 p17 subunit level on the background of declined procaspase-3 content in the area of the healthy tooth. At the same time, we obtained different results in the area of the impacted tooth and found a simultaneous elevation of both procaspase-3 and caspase-3 p17 subunit. According to these findings, a ratio between subunit p17 and procaspase-3 was lower in the area of the impacted tooth compared with the bone tissue, which was taken near the healthy tooth in patients with tooth impaction. These observations indicate the enhanced cleavage of procaspase-3 and its activation in the area of healthy tooth of patients with IT, that promote the tooth eruption process. We have to emphasize that the impairment of caspase-3 activation and the accumulation of inactive form in the area of tooth impaction may lead to tooth eruption failure. Another evidence of impaired or even inhibited apoptosis in the IT area may be elevated OPG level, since it has been proven that OPG inhibits apoptosis [26]. In addition, it may be assumed that elevated caspase-3 level in the impaction area may possibly indicate the death of functional cells – preosteoclasts and osteoblasts, and explain the absence of NF-κB activation after the observed activation of the RANKL/RANK/OPG system near the IT.

The obtained experimental results may be beneficial for practical medicine, especially for the new field of molecular orthodontics, as these data extend the current understanding of the cellular and molecular mechanisms of tooth impaction in humans and emphasize the role of the NF-κB-associated RANKL/RANK/OPG signaling pathway in disturbances of tooth eruption. Based on our data, therapeutic approaches for patients with failed tooth eruption should be directed to restoring the normal course of the resorption process in order to facilitate tooth eruption. Thus, to improve the functioning of local cytokine pathways responsible for osteoregulation in patients with impacted teeth, mechanical stimulation for local therapy may be beneficial. A removable orthodontic apparatus, developed earlier by Flis et al. [27] allows improving blood circulation within the area of the impacted tooth that may influence the state of molecular pathways involved in bone remodeling. The therapeutic use of this apparatus could stimulate mechanically the formation of an eruptive pathway, set the direction for a mature impacted tooth with high cellular potential, and facilitate its movement to the oral cavity.

## Conclusions

Our findings suggest that tooth impaction may be associated with blocked NF-κB signaling, disturbances in the cascade activation of caspase-3, and the imbalance in the ratio of components of the RANKL/RANK/OPG signaling axis, and as a consequence, the partially blocked or delayed process of bone resorption. The results of the study may be taken into account in developing treatment protocols for patients with impacted teeth and contribute to the field of molecular orthodontics.

## Supplementary information

**Additional file 1.**

## Data Availability

The data that support the findings of this study are partially available in Supplementary Information file but restrictions apply to the availability of these data, which were used under license of a research grant No. 0119 U101229 for the current study, and so are not fully publicly available. Data are however available from the corresponding author upon reasonable request and with permission of the Ethics and Research Committee of Bogomolets National Medical University and Dr. Natrus L.V., a Chief Grant Management Officer.

## Abbreviations

IT: Impacted tooth
M-CSF 1: Macrophage colony-stimulating factor 1
NFATc1: Nuclear factor of activated T-cells 1
NF-κB: Nuclear factor kappa B
OC: Osteocalcin
OPG: Osteoprotegerin
RANK: Receptor activator of NFκB
RANKL: Receptor activator of NF-κB ligand
TNFα: Tumor necrosis factor alpha
