# Supplementary material for: The regulatory role of the RANKL/RANK/OPG signaling pathway in the mechanisms of tooth eruption in patients with impacted teeth
Source: BMC Oral Health. 2020 Sep 18;20:261. doi: 10.1186/s12903-020-01251-y (PMC7501598; doi:10.1186/s12903-020-01251-y)
Supplement: Supplementary file 1 — Additional file 1. [file 12903_2020_1251_MOESM1_ESM.pdf]

Supplementary Information file

Initial set of Western blot experiments

(full-length, original, unprocessed versions of blots)

Table 1 Sample labelling

Patients	Group	Patient №	Sample labelling	Weight of bone tissue sample, g
Patients with normally erupted teeth	#1 – bone tissue, that was taken from the area of the normally erupted third molar (control group)	1	**1-1**	0.0457
		3	**1-2**	0.0522
		4	**1-3**	0.0674
		5	**1-4**	0.0575
		6	**1-5**	0.0661
		8	**1-6**	0.0355
Patients with tooth impaction	#2 – bone tissue of patients with tooth impaction, collected from the area of the normally erupted third molar	9	**2-1**	0.0341
		10	**2-2**	0.0481
		11	**2-3**	0.0452
		12	**2-4**	0.0503
		13	**2-5**	0.0388
		14	**2-6**	0.0426
	#3 – bone tissue of patients with tooth impaction, collected from the area of the impacted third molar	15	**3-1**	0.0513
		16	**3-2**	0.0641
		17	**3-3**	0.0852
		18	**3-4**	0.0374
		19	**3-5**	0.0371
		20	**3-6**	0.0509

Fig. 1 Raw data from Western blot experiments using RANK antibody

Immunoblotting was conducted with primary antibody against RANK (1:500; sc-9072, Santa Cruz, USA). Samples are labelled (according to the Table 1) under the images.

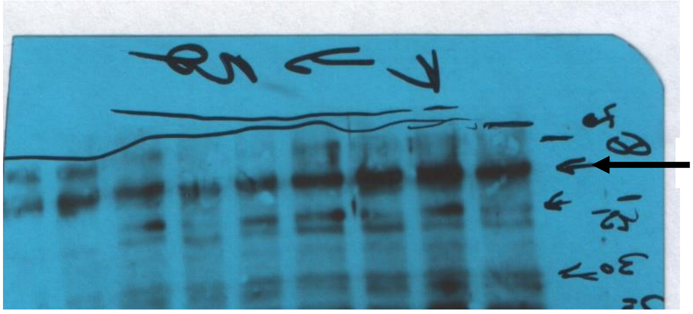

RANK, 66 kDa

1-1 1-2 1-3 2-1 2-2 2-3 3-1 3-2 3-3

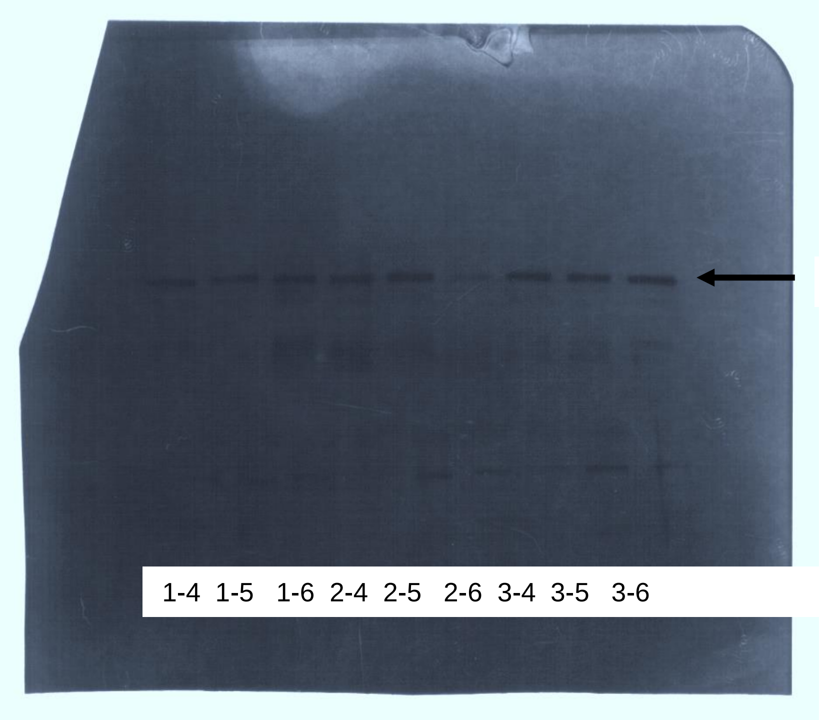

RANK, 66 kDa

Fig. 2 Raw data from Western blot experiments using osteoprotegerin (OPG) antibody

Immunoblotting was conducted with primary antibody against OPG (1:250; sc-8468, Santa Cruz, USA). Samples are labelled (according to the Table 1) under the image.

10 sec exposition

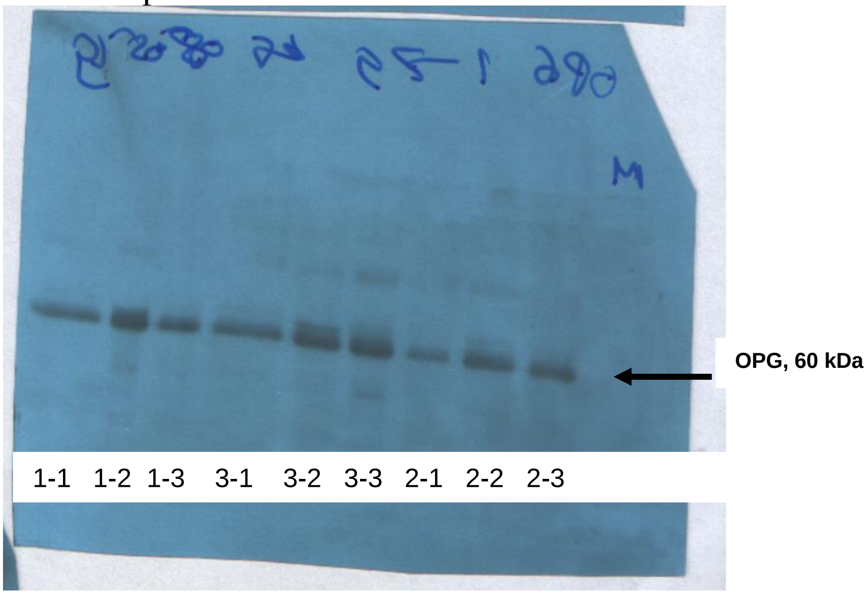

30 sec exposition

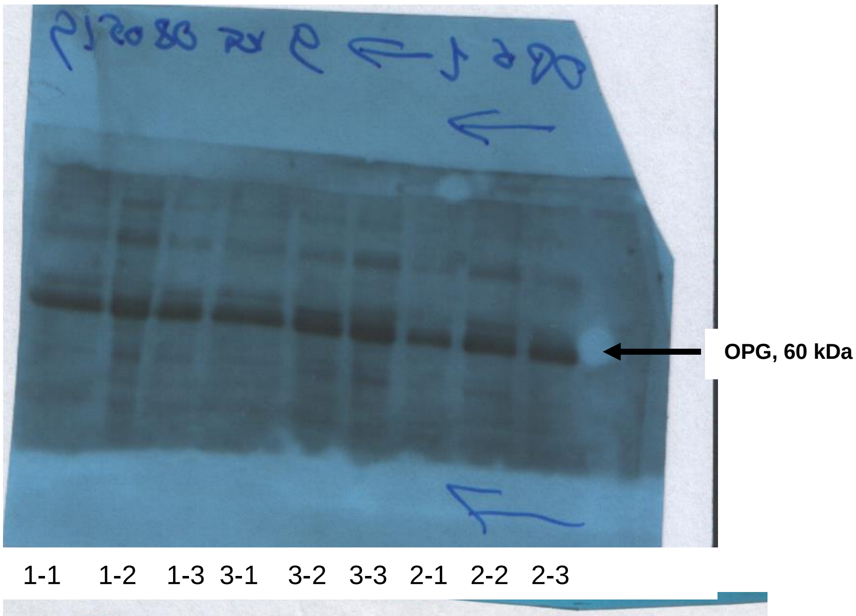

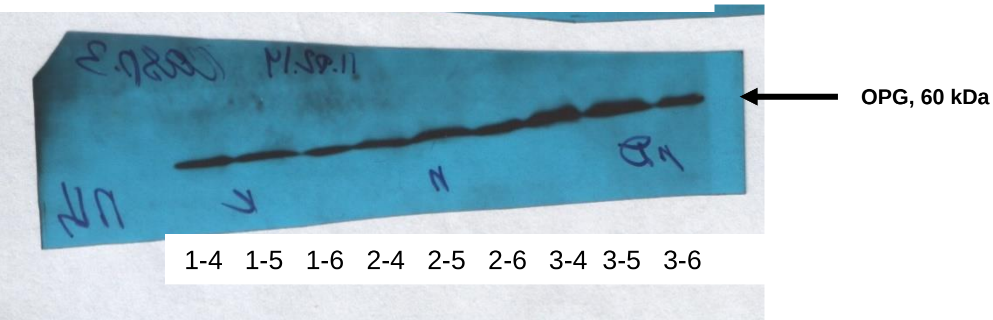

Fig. 3 Raw data from Western blot experiments using osteocalcin (OC) antibody

Immunoblotting was conducted with primary antibody against OC (1:500; sc-18319, Santa Cruz, USA). Samples are labelled (according to the Table 1) under the image.

10 sec exposition

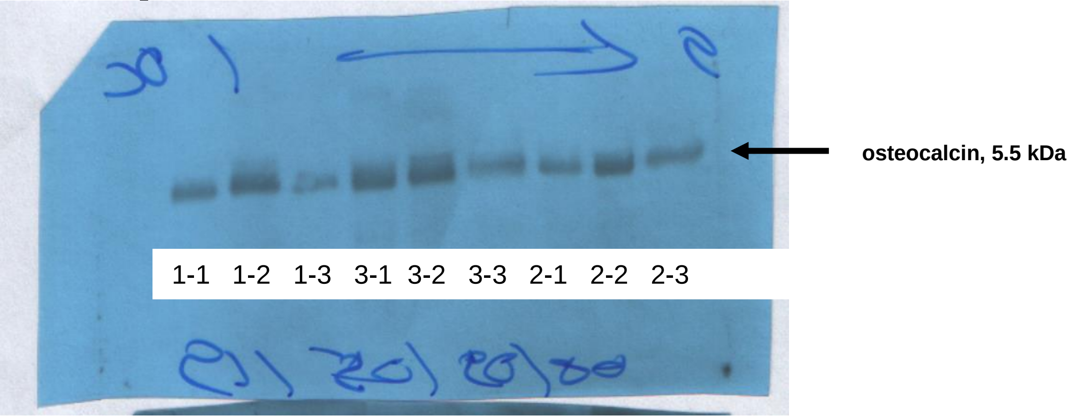

25 sec exposition

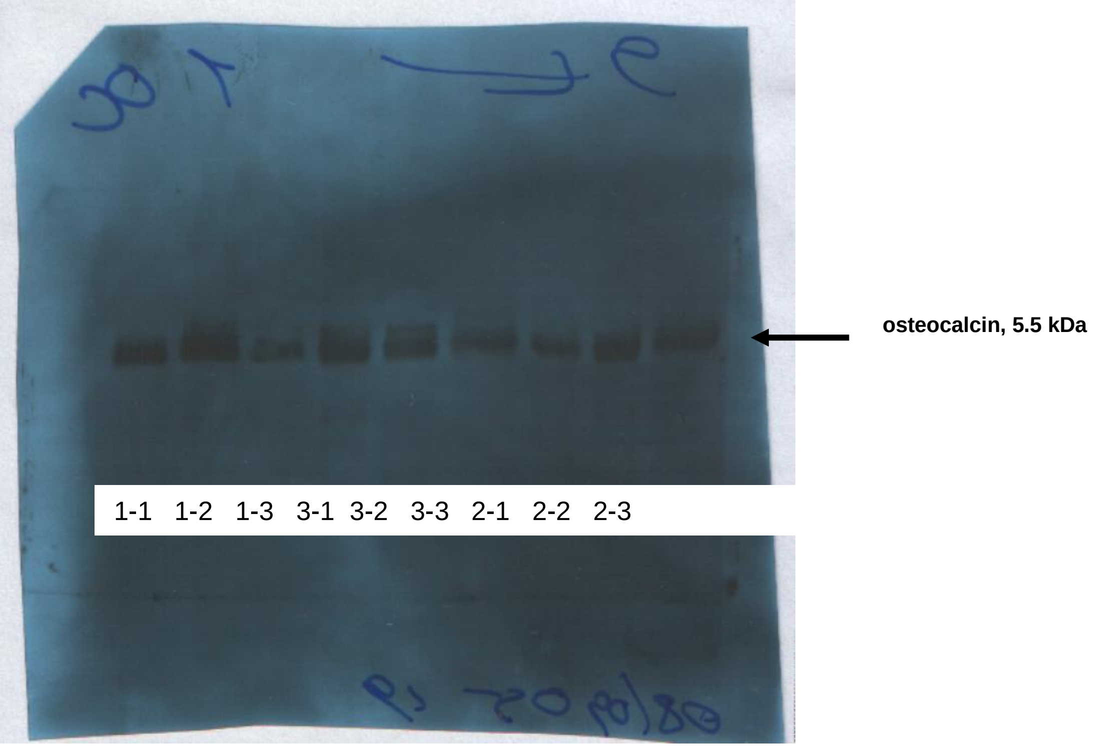

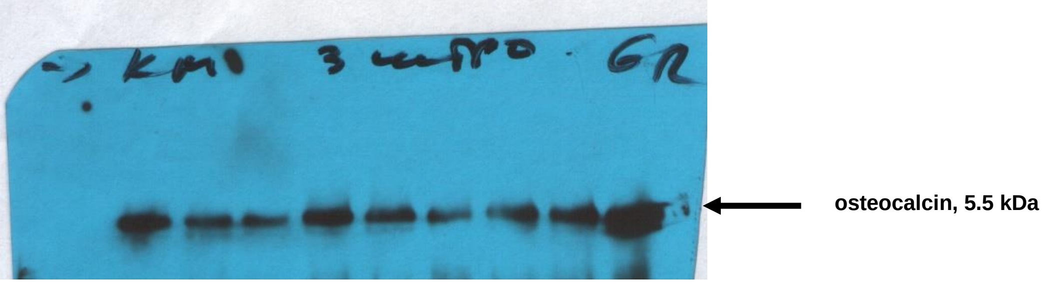

1-4 1-5 1-6 2-4 2-5 2-6 3-4 3-5 3-6

Fig. 4 Raw data from Western blot experiments using RANKL antibody

Immunoblotting was conducted with primary antibody against RANKL (1:250; sc-7628, Santa Cruz, USA). Samples are labelled (according to the Table 1) under the image.

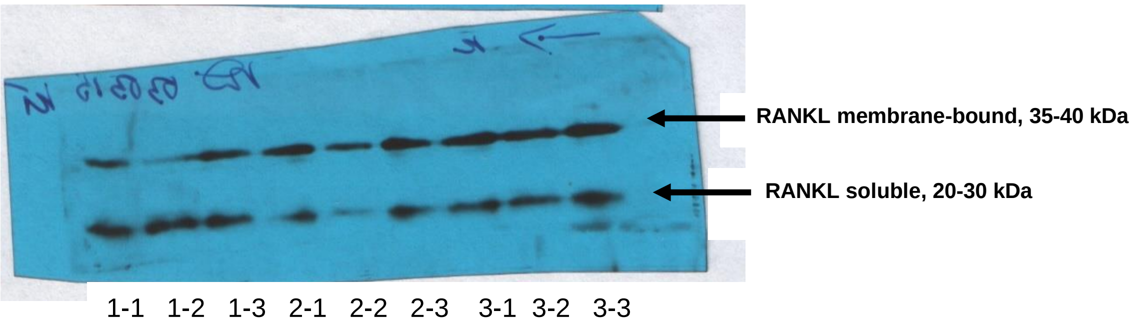

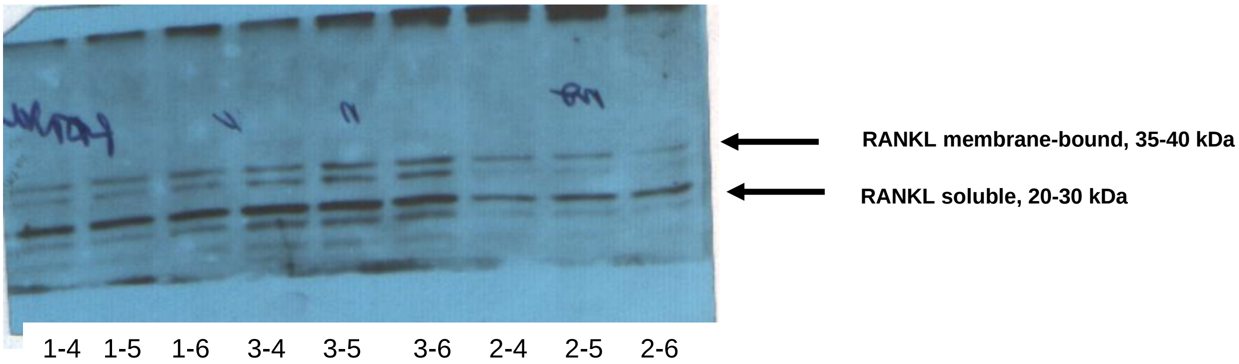

Fig. 5 Raw data from Western blot experiments using NF-κB antibody

Immunoblotting was conducted with primary antibody against NF-κB p65 subunit (1:500; MA5-16160, Thermo Fisher Scientific, USA). Samples are labelled (according to the Table 1) under the image.

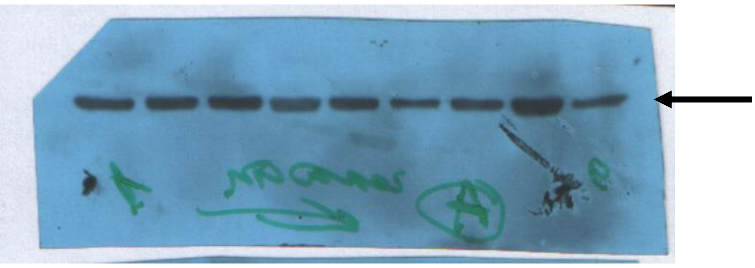

NF-κB, 65 kDa

1-1 1-2 1-3 2-1 2-2 2-3 3-1 3-2 3-3

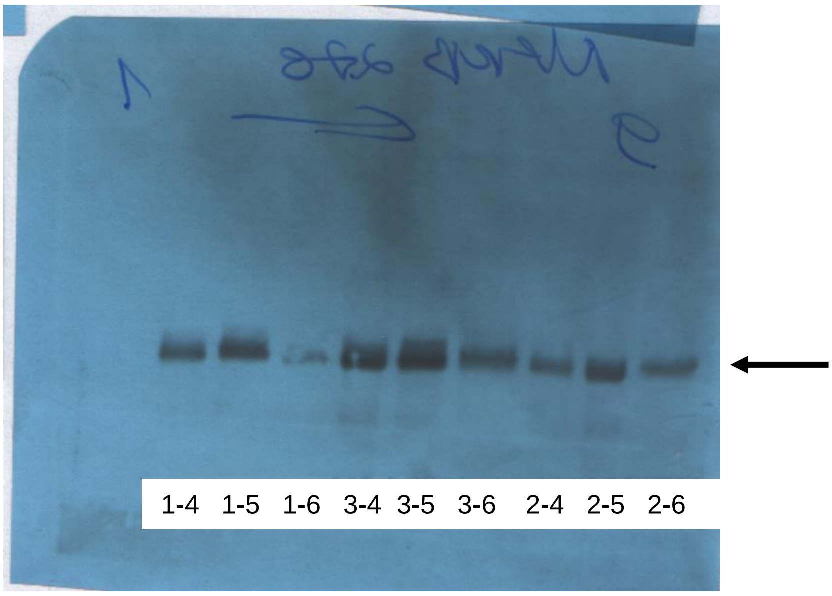

NF-κB, 65 kDa

Fig. 6 Raw data from Western blot experiments using NFATc1 antibody

Immunoblotting was conducted with primary antibody against NFATc1 (1:1000; MA3-024, Thermo Fisher Scientific, USA). Samples are labelled (according to the Table 1) under the image.

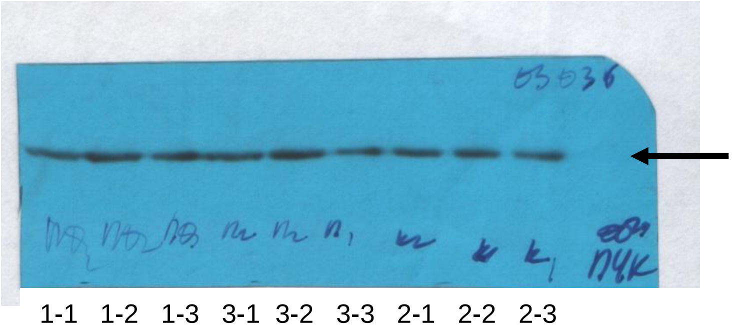

NFATc1, 105 kDa

1-1 1-2 1-3 3-1 3-2 3-3 2-1 2-2 2-3

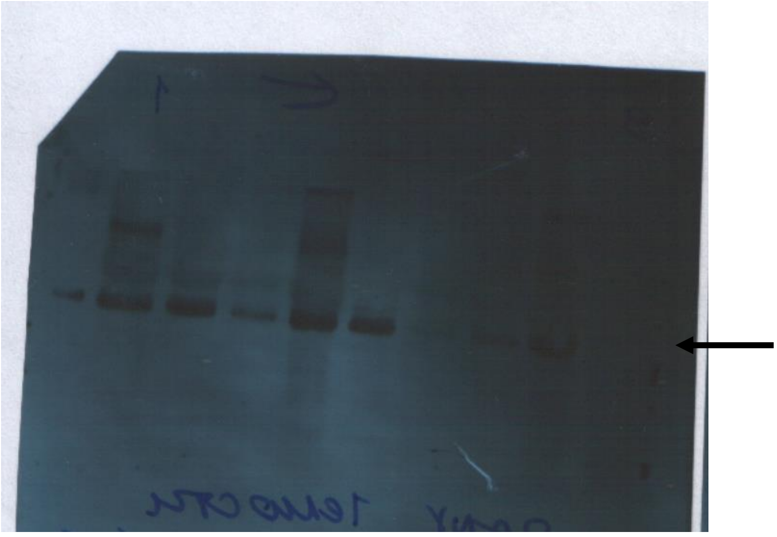

NFATc1, 105 kDa

1-4 1-5 1-6 3-4 3-5 3-6 2-4 2-5 2-6

Fig. 7 Raw data from Western blot experiments using caspase-3 antibody

Immunoblotting was conducted with primary antibody against caspase-3 (1:500; sc-373730, Santa Cruz, USA). Samples are labelled (according to the Table 1) under the image.

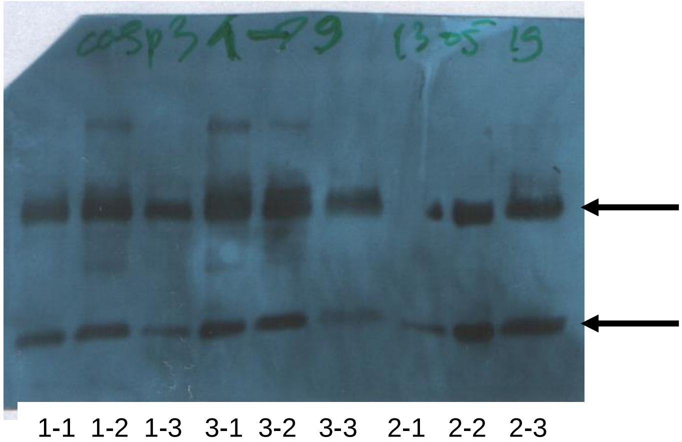

procaspase-3, 32 kDa

caspase-3 p17, 17 kDa

1-1 1-2 1-3 3-1 3-2 3-3 2-1 2-2 2-3

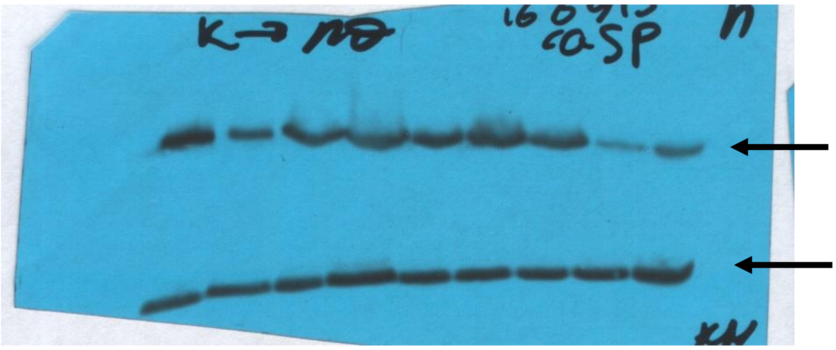

procaspase-3, 32 kDa

caspase-3 p17, 17 kDa

1-4 1-5 1-6 3-4 3-5 3-6 2-4 2-5 2-6
